# White-Nose Syndrome Fungus (*Geomyces destructans*) in Bats, Europe

**DOI:** 10.3201/eid1608.100002

**Published:** 2010-08

**Authors:** Gudrun Wibbelt, Andreas Kurth, David Hellmann, Manfred Weishaar, Alex Barlow, Michael Veith, Julia Prüger, Tamás Görföl, Lena Grosche, Fabio Bontadina, Ulrich Zöphel, Hans-Peter Seidl, Paul M. Cryan, David S. Blehert

**Affiliations:** Leibniz Institute for Zoo and Wildlife Research, Berlin, Germany (G. Wibbelt); Robert Koch Institute, Berlin (A. Kurth); University of Oldenburg, Oldenburg, Germany (D. Hellmann); Bat Conservation Working Group, Gusterath, Germany (M. Weishaar); Veterinary Laboratory Agency, Somerset, UK (A. Barlow); Trier University, Trier, Germany (M. Veith); Coordination Agency for Bat Protection in Thuringia, Erfurt, Germany (J. Prüger); Nature Conservation Foundation of Tolna County, Szekszárd, Hungary (T. Görföl); Echolot GbR, Münster, Germany (L. Grosche); SWILD–Urban Ecology and Wildlife Research, Zurich, Switzerland (F. Bontadina); Saxonian State Office for Environment, Agriculture and Geology, Dresden-Pillnitz, Germany (U. Zöphel); Technical University Munich, Munich, Germany (H.-P. Seidl); US Geological Survey, Fort Collins, Colorado, USA (P.M. Cryan); US Geological Survey, Madison, Wisconsin, USA (D.S. Blehert)

**Keywords:** White-nose syndrome, Geomyces destructans, bats, Europe, fungi, mortality, pathogen, hibernation, Myotis spp., research

## Abstract

White-nose syndrome is an emerging disease in North America that has caused substantial declines in hibernating bats. A recently identified fungus (*Geomyces destructans*) causes skin lesions that are characteristic of this disease. Typical signs of this infection were not observed in bats in North America before white-nose syndrome was detected. However, unconfirmed reports from Europe indicated white fungal growth on hibernating bats without associated deaths. To investigate these differences, hibernating bats were sampled in Germany, Switzerland, and Hungary to determine whether *G. destructans* is present in Europe. Microscopic observations, fungal culture, and genetic analyses of 43 samples from 23 bats indicated that 21 bats of 5 species in 3 countries were colonized by *G. destructans.* We hypothesize that *G. destructans* is present throughout Europe and that bats in Europe may be more immunologically or behaviorally resistant to *G. destructans* than their congeners in North America because they potentially coevolved with the fungus.

## Abstract 2

Unlike bats in North America, bats in Europe are not killed by this fungus.

White-nose syndrome (WNS) is a recently emerged wildlife disease in North America, which in 4 years has resulted in unprecedented deaths of hibernating bats in the northeastern United States (*1**–**3*), and is a widespread epizootic disease among bats. Although we have searched the literature describing observations of hibernating bats, we have been unable to find any similar historical accounts of white fungus growing on live hibernating bats in North America before the recent emergence of WNS.

In North America, WNS is known to affect 6 species of bats that use hibernation as their winter survival strategy: the big brown bat (*Eptesicus fuscus*), the eastern small-footed bat (*Myotis leibii*), the little brown bat (*M. lucifugus*), the northern long-eared bat (*M. septentrionalis*), the tricolored bat (*Perimyotis subflavus*), and the Indiana bat (*M. sodalis*) (*1**,**3**,**4*). Since its detection in February 2006 in a popular tourist cave near Albany, New York, USA, WNS has spread >1,300 km into Connecticut, Massachusetts, New Hampshire, New Jersey, Pennsylvania, Tennessee, Vermont, Virginia, and West Virginia in the United States and the provinces of Ontario and Quebec in Canada (*1**,**3**,**5*) in a pattern suggesting the spread of an infectious agent.

A recently discovered psychrophilic (cold-loving) fungus, *Geomyces destructans* (*6*), has consistently been isolated from bats that meet the pathologic criteria for WNS, including colonization of skin by fungal hyphae causing characteristic epidermal erosions and ulcers that can progress to invasion of underlying connective tissue (*2**,**7*). *G*. *destructans* is identified by its distinctive asymmetrically curved conidia and has a unique taxonomic position among other *Geomyces* spp. described to date (*6*). Its closest genetic relative is *G. pannorum*, a ubiquitous psychrophilic, keratinolytic fungus that has been isolated from a variety of sources and geographic regions, including soil and the fur of wild mammals in France (*8*), floors of trains and ferryboats in Italy (*9*), boreal forests in Canada (*10*), and environmental samples from Arctic regions (*11**,**12*). *G. pannorum* var. *pannorum* has been reported as an unusual dermatophyte infecting fingernails and superficial skin of humans who have a history of close contact with soil and dust (*13**,**14*). However, *G. destructans* differs from other common soil fungi of North America in its ability to invade the living tissues of hibernating bats.

After WNS was described in North America (*1*), reports dating back to the early 1980s (*15*) described repeated observations of white fungal growth on muzzles of hibernating bats in Germany. However, these bats lacked the characteristics of WNS such as associated deaths. Moreover, fungus was not identified. In response to WNS in North America, researchers in Europe initiated a surveillance effort during the winter of 2008–09 for WNS-like fungal infections among hibernating populations of bats in Europe. *G. destructans* in Europe was previously reported in 1 hibernating bat that was sampled in France during March 2009 (*16*).

In this report, we describe results of a more extensive effort by scientists from 4 countries in Europe (Germany, United Kingdom, Hungary, and Switzerland) to obtain and analyze samples from hibernating bats with white patches on their faces or wing membranes. Our objectives were to identify the fungus colonizing such affected hibernating bats in Europe and to clarify its geographic distribution over a broad area of Europe.

## Materials and Methods

**Figure 1:**
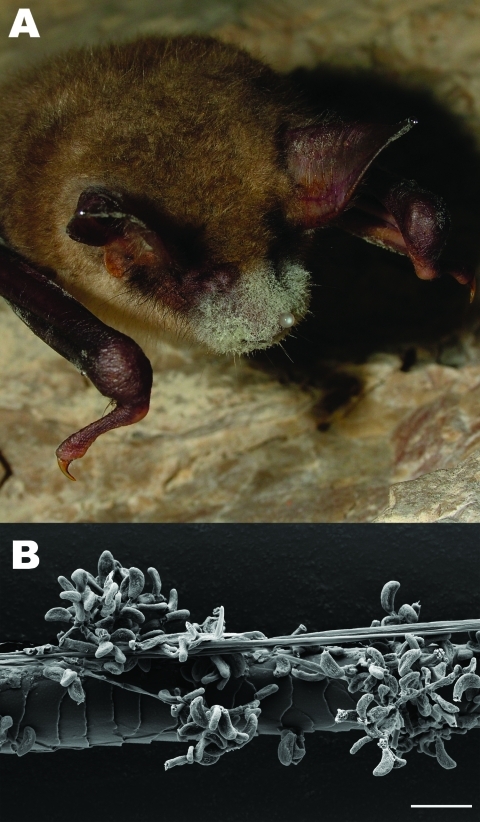
A) Greater mouse-eared bat (*Myotis myotis*) with white fungal growth around its muzzle, ears, and wing membranes (photograph provided by Tamás Görföl). B) Scanning electron micrograph of a bat hair colonized by *Geomyces destructans*. Scale bar = 10 µm.

During ongoing annual population surveys of caves and mines conducted by national nongovernmental organizations, hibernating bats with obvious fungal growth on their bodies (Figure 1, panel A) were opportunistically sampled in Germany, Switzerland, and Hungary; samples were also obtained from 2 dead bats from the same hibernaculum in the United Kingdom. Approximately 366 hibernacula were visited during mid-February–mid-April 2009: 336 in Germany, 20 in Hungary, and 10 in Switzerland. Two to 214 hibernating animals were observed at each site, with the exception of 2 sites in Germany, which harbored 2,000–7,000 animals at each site.

Samples were collected from live bats by using 2 methods. Touch imprints were obtained by holding adhesive tape against affected areas of skin or fur, or fur clippings were obtained from affected areas of bat muzzles. All species of bats in Europe are strictly protected under the Flora, Fauna, Habitat Guidelines of the European Union (92/43/EEC) (http://ec.europa.eu/environment/nature/legislation/habitatsdirective/index_en.htm) and The Agreement on the Conservation of Populations of European Bats (www.eurobats.org). We did not have permission to invasively sample or kill individual animals for histologic analysis to confirm skin infection by *G. destructans* (*7*). Samples were shipped to the Leibniz Institute of Zoo and Wildlife Research (IZW), Berlin, Germany, for further investigations.

Twenty adhesive tape samples were first screened by using light microscopy, and 21 hair samples were examined by using scanning electron microscopy for conidia characteristic of *G. destructans* (Figure 1, panel B). Two of the submitted samples (2 greater horseshoe bats from the United Kingdom) consisted of entire bat carcasses. Although the carcasses were examined externally for fungal growth on muzzle skin and hair, specimens were too decomposed to conduct internal pathologic examinations. Tape or hair samples from all bats were further investigated by using direct PCR amplification of fungal rRNA gene internal transcribed spacer (ITS) region DNA (ITS1, 5.8S, and ITS2). Total nucleic acids were extracted from culture, tape, or hair samples by using PrepMan Ultra Reagent (Applied Biosystems, Darmstadt, Germany) following the manufacturer’s instructions.

The rRNA gene ITS region DNA was amplified by using PCR with primers ITS4 and ITS5 (*17*) and GoTaq DNA polymerase (Promega, Madison, WI, USA). Cycling parameters were an initial 2-min denaturation at 98°C; followed by 30 cycles of denaturation at 98°C for 10 s, annealing at 50°C for 30 s, and extension at 72°C for 1 min; and a final extension at 72°C for 7 min. For fungal isolates, rRNA gene small subunit (SSU) DNA was amplified by using PCR with primers nu-SSU-0021–5′ (*17*) and nu-SSU-1750–3′ (*18*) as above, except the extension time was increased to 2 min. Sequencing primers were PCR primers; nu-SSU-0402–5′ (*18*), nu-SSU-1150–5′ (*17*), nu-SSU-0497–3′ (*18*), and nu-SSU-1184–3′ (*19*) were added for SSU. PCR products were sent to the Robert Koch Institute, Berlin, Germany, for direct sequencing.

Culture analyses of samples were performed at Munich University Hospital and IZW. After examining tape impressions by using light microscopy, we identified small areas with fungal conidia characteristic of *G*. *destructans* and excised them with a sterile scalpel blade. Half of the excised material was used for PCR; the remaining sample and samples of individual hairs with microscopic indication of *G. destructans* were immediately placed onto Sabouraud dextrose agar plates containing gentamicin and chloramphenicol and incubated at 4°C and 8°C. *G*. *destructans* isolates obtained during this study are maintained at IZW.

## Results

We obtained and analyzed samples from live bats with obvious fungal growth on their bodies found between mid-February and the end of March 2009 at 11 sites (8 in Germany, 1 in Hungary, and 2 in Switzerland). Samples were also obtained from an additional bat in Germany in February 2008 and from 2 dead bats from a site in the United Kingdom in March 2009 (Tables 1, 2) All 12 hibernacula sampled contained 1–5 animals that exhibited obvious fungal growth. Forty-three samples were obtained from these 12 hibernacula and represented 23 adult bats of 6 species: 1 Brandt bat (*M. brandtii*), 3 pond bats (*M. dasycneme*), 1 Daubenton bat (*M. daubentonii*), 1 lesser mouse-eared bat (*M. oxygnathus*), 15 greater mouse-eared bats (*M. myotis*), and 2 greater horseshoe bats (*Rhinolophus ferrumequinum*).

**Table 1:** Bats tested for *Geomyces destructans* by using microscopy, fungal culture, or PCR analysis, by country, Europe*

Species (common name)	No. positive/no. tested			
	Germany	Switzerland	Hungary	United Kingdom
*Myotis myotis* (greater mouse-eared bat)	10/10	4/4	1/1	–
*M. dasycneme* (pond bat)	3/3	–	–	–
*M. daubentonii* (Daubenton bat)	1/1	–	–	–
*M. brandtii* (Brandt bat)	1/1	–	–	–
*M. oxygnathus* (lesser mouse-eared bat)	–	­–	1/1	–
*Rhinolophus ferrumequinum* (greater horseshoe bat)	–	–	–	0/2

*–, species not obtained in this country.

**Table 2:** Fungal culture and PCR results for 23 bats with evidence of fungal colonization tested by light or electron microscopy, Europe*

Country/ location no.†	Sample source	Species	Collection date	No./ hibernacula	PCR result	Culture result	GenBank accession no.	
							ITS	SSU rRNA
Germany/4	Hair 2	*Myotis dasycneme*	2008 Feb 25	10	+	+	GU350437	GU350442
Germany/8	Hair 7	*M. myotis*	2009 Mar 3	214	+	+	GU350436	GU350441
Germany/7	Tape 8	*M. myotis*	2009 Mar 7	57	+	+	GU999986	GU999983
Hungary/9	Hair 16	*M. myotis*	2009 Mar 29	64	+	+	GU350434	GU350439
Switzerland/10	Tape 10	*M. myotis*	2009 Apr 5	25	+	+	GU350433	GU350438
Switzerland/10	Tape 11	*M. myotis*	2009 Apr 5	25	+	+	GU999984	GU999981
Switzerland/11	Tape 12	*M. myotis*	2009 Apr 5	25	+	+	GU999985	GU999982
Switzerland/10	Tape 20	*M. myotis*	2009 Apr 11	25	+	+	GU350435	GU350440
Germany/1	Hair 1	*M. myotis*	2009 Feb 21	65	+	–	HM222616	–
Germany/6	Hair 20	*M. myotis*	2009 Mar 13	100	+	–	HM222617	–
Germany/2	Tape 1	*M. myotis*	2009 Feb 26	≈2,000	+	–	HM222618	–
Germany/2	Tape 2	*M. myotis*	2009 Feb 26	≈2,000	+	–	HM222619	–
Germany/8	Tape 5	*M. myotis*	2009 Mar 3	214	+	–	HM222620	–
Germany/8	Tape 6	*M. myotis*	2009 Mar 3	214	+	–	HM222621	–
Germany/7	Tape 9	*M. myotis*	2009 Mar 7	57	+	–	HM222622	–
Germany/6	Tape 16	*M. myotis*	2009 Mar 13	100	+	–	HM222623	–
Germany/8	Hair 6	*M. brandtii*	2009 Mar 3	214	+	–	HM222624	–
Germany/5	Hair 3	*M. dasycneme*	2009 Feb 28	29	+	–	HM222625	–
Germany/6	Tape 17	*M. dasycneme*	2009 Mar 13	100	+	–	HM222626	–
Germany/3	Hair 17	*M. daubentonii*	2009 Mar 5	≈7,000	+	–	HM222627	–
Hungary/9	Tape 13	*M. oxygnathus*	2009 Mar 29	64	+	–	HM222628	–
United Kingdom/12	Hair 10	*Rhinolophus ferrumequinum*	2009 Mar 11	558	–‡	–‡	HM222629	–
United Kingdom/12	Hair 11	*R. ferrumequinum*	2009 Mar 11	558	–‡	–‡	HM222630	–

**Figure 2:**
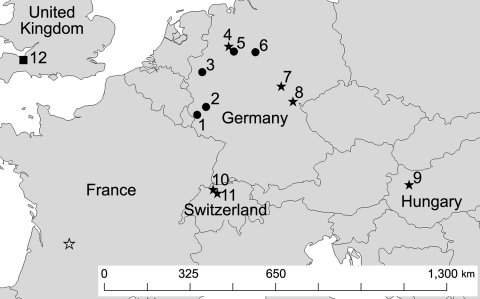
Locations in Europe of bats positive for *Geomyces destructans* by PCR alone (circles) or by PCR and culture (solid stars) and bats negative for *G. destructans* but positive for other fungi (square). Numbers for locations correspond to those in Table 2. Sites 7, 8, and 9 had additional bats that were positive for *G. destructans* only by PCR. Location of a bat positive for *G. destructans* in France (*16*) is indicated by an open star. Some sites had >1 bat species with evidence of colonization by *G.* *destructans*.

*ITS, internal transcribed spacer; SSU, small subunit; tape, touch imprint with adhesive tape. †Location number corresponds to a hibernation site in Figure 2. ‡Although samples were negative for *G. destructans*, they were positive for *Penicillium* sp. by PCR and culture.

After direct PCR amplification and DNA sequence analysis of fungal rRNA gene ITS regions, genetic signatures 100% identical with those from *G*. *destructans* type isolate NWHC 20631–21 (GenBank accession no. EU884921) were identified from 21 of 23 bats examined: 15/15 from Germany, 2/2 from Hungary, and 4/4 from Switzerland. Both bats from the United Kingdom were colonized by *Penicillium* sp. (Tables 1, 2). Fungi with conidia morphologically identical to those of *G*. *destructans* (Figure 1, panel B) as described by Gargas et al. (*6*) were isolated in axenic cultures from 8 of 23 bats examined: 3/15 from Germany, 1/2 from Hungary, and 4/4 from Switzerland) (Tables 1, 2; Figure 2).

Consistent with published descriptions for *G*. *destructans* (*6*), fungal colonies grew slowly and within 14 days attained diameters of 1.0 mm at 4°C and 4.0–5.0 mm at 8°C; no growth occurred at 25°C. The sensitivity of our method for isolating *G*. *destructans* from bat hair was comparable to published diagnostic sensitivity for culturing *G*. *destructans* from bat skin (*20*). Subsequent PCR/DNA sequencing analyses of the 8 isolates indicated that they all had rRNA gene ITS and SSU region DNA sequences identical to those of *G*. *destructans* type isolate NWHC 20631–21 (GenBank accession nos. EU884921 for ITS and FJ231098 for SSU).

Unlike other bats sampled in this study, the 2 greater horseshoe bats from the United Kingdom were found dead, and their nostrils were colonized by *Penicillium* sp. These bats did not fulfill the pathologic criteria for WNS (*7*) because fungal hyphae did not invade the epidermis but remained within the superficial layer of the epidermal stratum corneum. A more complete description of the postmortem analysis of the greater horseshoe bats has been reported (*21*). *G*. *destructans* was not isolated in culture, and its genetic signature was not identified by PCR and DNA sequencing of samples collected from greater horseshoe bats.

## Discussion

Laboratory analyses demonstrated that 5 species of the genus *Myotis* in Europe harbored *G*. *destructans*; male and female bats were equally affected. Despite laboratory confirmation that bats obtained in Germany, Switzerland, and Hungary were colonized by *G*. *destructans*, deaths were not observed at collection sites. Puechmaille et al. (*16*) reported a similar observation with a greater mouse-eared bat in France. Additionally, a lesser mouse-eared bat from Hungary with visible fungal infection during hibernation, from which *G*. *destructans* was isolated, was recaptured 5 months later (August 2009) and showed no external signs of fungal infection. On February 19, 2010, the same bat was again observed in the same hibernaculum without any visible sign of fungal growth. However, 7 other bats within that group of 55 animals displayed obvious fungal growth but were not sampled for this study.

In contrast, decreases in bat hibernating colonies infected by *G*. *destructans* in North America are often >90% (*2**,**3*), and mortality rates similar in magnitude would be difficult to miss among closely monitored winter populations of bats in Europe. Biologists in Germany and Switzerland have conducted annual censuses of bat hibernacula since the 1930s and 1950s, respectively. In Hungary, the largest hibernacula have been annually monitored since 1990. Similar death rates to those caused by WNS in hibernating bats in North America have never been documented in countries in Europe in which *G*. *destructans* has now been identified.

Although distribution of *G*. *destructans* in bats across Europe has not been exhaustively characterized, opportunistic sampling conducted as part of this study during the winter of 2008–09 demonstrated that the fungus was present on bats in 3 countries (Figure 2). The 2 most distant points from which bats colonized with *G*. *destructans* have been identified were separated by >1,300 km. Despite the observed distribution of *G*. *destructans* in Europe (Figure 2), the 5 bat species from which *G. destructans* was detected migrate average distances <100 km between their summer and winter roosting sites (*22*), indicating that the fungus is most likely spread as local bat populations emerge from hibernacula, disperse, and interact with populations within their dispersal range. Identification of bats colonized by *G*. *destructans* from such distant sites, in addition to the relatively homogenous distribution of the fungus among sites in Germany, suggests that *G*. *destructans* may be widespread in Europe.

Regardless of widespread occurrence of *G*. *destructans* among bat species in Europe (Figure 2), deaths of bats in Europe caused by WNS, similar to those caused by WNS in North America, have not been observed. Although no bat species migrates between Europe and North America or is present on both continents (*23**,**24*), many species of the genus *Myotis* are infected by *G*. *destructans* on each continent. Although the mechanism(s) by which hibernating bats died because of infection with *G. destructans* in North America is not yet understood, bat species in Europe may exhibit greater resistance or respond differently to infection by this fungus than their counterparts in North America.

Before the emergence of WNS in North America, large aggregations of hibernating bats ranging from 1,000 to 50,000 animals were common in caves and mines of affected regions, and many hibernation sites in regions of North America still unaffected by WNS contained tens of thousands of bats during winter (some contain hundreds of thousands) (*25*). In contrast, aggregations of bats hibernating in caves and mines in Europe rarely exceed 1,000 animals. However, larger hibernating groups have been observed at a few natural sites, such as a cave in northern Germany with 13,000–18,000 bats (*26*) and human-made structures (e.g., Daubenton bats in bunkers and catacombs) (*24*). If host density plays a role in *G*. *destructans* transmission or deaths of bats, such as through increased disturbance of clustered bats, the bats in Europe may experience lower mortality rates because they form smaller hibernation groups composed of small clusters or individual bats. Apparent continental differences in susceptibility of hibernating bats to deaths associated with skin infection by *G*. *destructans* may indicate either circumstantial or evolved resistance in bats in Europe.

*G*. *destructans* has been detected in North America only in states and provinces where WNS has also been observed and in contiguous states. Recent emergence and spread of *G*. *destructans* with associated deaths of bats throughout hibernacula in the northeastern United States (*3*) may suggest ecologic release of an exotic pathogen into an uninfected ecosystem. Although this suggestion remains a hypothesis and how *G*. *destructans* may have been introduced to the United States is not known, initial documentation of WNS in a popular tourist cave near Albany, New York (*1*), suggests that a human vector could have been involved.

There are many examples of unintended introductions of fungal pathogens, particularly of those affecting plants and ectothermic animals with tissue temperatures permissive to fungal infection (*27**–**29*). One case with striking similarities is the panzootic chytrid fungus (*Batrachochytrium dendrobatidis*), which has caused global decreases among amphibian species (*30*). As with skin infection by *B*. *dendrobatidis* in amphibians, which can alter body electrolyte levels and lead to cardiac arrest (*31*), skin infection by *G*. *destructans* in hibernating bats may also kill by causing irreversible homeostatic imbalance because wing membranes play major roles in water balance, circulation, and thermoregulation of hibernating bats during winter (*32**,**33*).

Bat species in Europe may be immunologically or behaviorally resistant to *G*. *destructans* because of having coevolved with the fungus. Additionally, microbial flora of bat skin or other abiotic surfaces in bat hibernacula in Europe may have also coevolved to incorporate *G*. *destructans* as a nonpathogenic component of the microbial community. Conversely, possible recent introduction of *G*. *destructans* into the United States, with subsequent infection of bat species in North America and ecosystems not infected with the fungus, provides a potential explanation for the devastating effects of WNS in North America. Although bats are reservoirs of various pathogens (*34**,**35*), research into the immune function of bats, particularly during hibernation, is just beginning.

In conclusion, nondetrimental colonization of bat species in Europe by *G*. *destructans* may be relatively common (Figure 2), and historical reports (*15*) suggest that such colonization of hibernating bats in Europe has occurred for several decades. In contrast to recent mass deaths associated with *G*. *destructans* skin infection, which is the hallmark of WNS in North America, bats in Europe appear to coexist with *G*. *destructans*. Studies to investigate mechanisms of pathogenesis, microbial ecology, and phylogeography of *G*. *destructans* will be essential for developing a comprehensive understanding of WNS. In particular, testing the hypotheses that bats in Europe are more resistant to fungal skin infection by *G*. *destructans*, that *G*. *destructans* was introduced from Europe to North America, and that environmental circumstances limit the pathogenicity of *G*. *destructans* in Europe seem warranted. Divergent manifestations of infection by *G*. *destructans* in bats in Europe and North America provide a unique opportunity to address these research objectives with the ultimate goals of better understanding WNS and developing sound strategies to manage the devastating effects of this emerging wildlife disease in North America.
